# Personalized Protocol for the Dynamic Assessment of Functional Biomarkers of Vascular Stiffness: A Novel Diagnostic Tool in P4 Medicine

**DOI:** 10.3390/diagnostics16132001

**Published:** 2026-06-26

**Authors:** Victor N. Dorogovtsev, Dmitry S. Yankevich, Valentina M. Tsareva, Denis A. Punin, Ilya V. Borisov, Julia A. Podolskaya, Andrey V. Grechko

**Affiliations:** 1 Laboratory of Innovative Rehabilitation Technologies, Federal Research and Clinical Center of Intensive Care Medicine and Rehabilitology, 107031 Moscow, Russia; dyankevich@fnkcrr.ru (D.S.Y.); iborisov@fnkcrr.ru (I.V.B.); upodolskaya@fnkcrr.ru (J.A.P.); noo@fnkcrr.ru (A.V.G.); 2 Department of Therapy, Ultrasound and Functional Diagnostics, Smolensk State Medical University, 214019 Smolensk, Russia; tsarev.al@mail.ru (V.M.T.); pun.92.work@gmail.com (D.A.P.)

**Keywords:** chronic age-related vascular diseases, risk-based prevention, P4 medicine, pulse wave velocity, head-up tilt test, orthostatic supine-to-sitting test, orthostatic changes in PWV, functional biomarker of vascular stiffness

## Abstract

**Background/Objectives:** Functional biomarkers of vascular stiffness (FBM-VS) may serve as an effective tool for predicting and monitoring the effectiveness of preventive strategies against accelerated vascular ageing in healthy populations within the framework of P4 medicine. The aim of this study was to perform a comparative analysis of a standardized to hydrostatic column height passive head-up tilt test (stHUTT) and a simplified supine-to-sitting test (SST) for measuring FBM-VS in a paired sample of young healthy subjects. **Materials and Methods:** This observational cross-sectional study included 95 healthy adults aged 18–20 years (54 women and 41 men). Brachial–ankle pulse wave velocity (baPWV) was measured in three positions: baseline supine position (baPWV_b_), during stHUTT (baPWV_st_), and after transitioning to a sitting position (baPWV_sit_). The functional reserve of orthostatic circulatory regulation (FR) and the functional reserve coefficient (FRC) were calculated for the stHUTT (FR_st_ and FRC_st_) and during the supine-to-sitting test (FR_sit_ and FRC_sit_). **Results:** The results showed unidirectional orthostatic changes in baPWV during both tests (significant increase compared to baseline supine values): baPWV_st_ and baPWV_sit_ in stHUTT and during the SST increased from 8.6 [8.1; 9.1] m/s to 13.4 [12.1; 14.4] m/s and to 15.2 [13.4; 16.1] m/s (*p* < 0.001), respectively. FBM-VS values in the SST were higher compared to stHUTT: FR_sit_ = 6.4 [5.25; 7.75] m/s vs. FR_st_ = 4.85 [3.7; 5.75] m/s (*p* < 0.001), and FRC_sit_ = 0.74 [0.59;0.9] vs. FRC_st_ = 0.55 [0.45; 0.68] (*p* < 0.001). The variance of these parameters was also significantly higher in the SST. Spearman rank correlation analysis demonstrated significant positive correlations between biomarkers measured during both orthostatic tests. **Conclusions:** The supine-to-sitting test may be used for the personalized quantitative assessment of FBM-VS in healthy populations. To assess their prognostic value and to provide personalized long-term monitoring to control the effectiveness of preventive measures against vascular ageing in healthy individuals, a prospective cohort study is required.

## 1. Introduction

The high prevalence of arterial hypertension (AH) and other age-related vascular diseases (ARVDs) remains a major public health challenge worldwide [1,2,3,4]. Moreover, an increasing trend in prevalence has been observed among younger populations [5,6]. Primordial and primary prevention of ARVDs are currently based on the management of well-established population-level risk factors [7,8,9,10,11,12]. Nevertheless, the upward trajectory in disease prevalence persists, and future projections remain concerning: prevalence is expected to continue rising, accompanied by substantial economic burdens [4,13]. This outlook underscores the need to enhance the efficacy of primary ARVDs prevention and to shift from reactive healthcare toward predictive and personalized approaches [14]. Whereas primary prevention targets individuals at high risk for vascular diseases, growing attention is being directed toward primordial prevention, which focuses on preventing the emergence of risk factors themselves [15]. This strategy appears both promising and feasible within the framework of P4 medicine [16,17,18]. Its core principles—prediction, prevention, participation and personalization—are increasingly being integrated into clinical practice [14,17,19]. In this context, it is of critical importance to predict the risk of developing age-related vascular diseases in apparently healthy populations and to implement preventive measures before these risks become clinically significant, i.e., in individuals who remain largely asymptomatic. It is well established that increased arterial stiffness represents a major independent risk factor for AH and other ARVDs [3,20,21,22,23]. Conventional assessment methods, typically performed under resting conditions in the supine position, allow for indirect evaluation of the status of the aortic and large arterial walls [24,25,26,27]. Such assessment of arterial stiffness, based on pulse wave velocity (PWV) analysis, predominantly reflects the structural rather than the functional component of stiffness. The functional component is highly dependent on neurohormonal status, which undergoes substantial changes in response to postural alterations in hydrostatic pressure [28,29,30]. We have proposed functional biomarkers of vascular stiffness (FBM-VS) designed to evaluate the adaptive capacity of the cardiovascular system in response to alterations in hydrostatic pressure induced by changes in body position [31,32]. For their assessment, we employed a head-up tilt test (Luanda Protocol) standardized to hydrostatic column height (stHUTT), which adjusted for inter-individual height differences and enabled more precise evaluation of orthostatic hemodynamic regulation [33]. Using this protocol, it was possible to determine the characteristics of FBM-VS in independent cohorts stratified by racial, gender and age subgroups [31,32,34].

However, the practical implementation of this approach necessitates the personalization of hydrostatic loading and, if possible, the elimination of the tilt table requirement, which renders the assessment of FBM-VS both logistically complex and economically infeasible. To address these limitations, we developed a simplified supine-to-sitting test (SST) that generates a personalized hydrostatic load in the sitting position, proportional to the subject’s height, without the need for bulky specialized equipment. Consequently, this simplified test enables the assessment of both resting PWV in the supine position, which primarily reflects the structural component of vascular stiffness, and the change in PWV during the test, which characterizes the adaptive capacity of the cardiovascular system. The aim of the study was a comparative analysis of the application of stHUTT and a simplified SST for measuring FBM-VS in a paired sample of healthy young subjects.

## 2. Materials and Methods

### 2.1. Study Design and the Selection of Participants

**Figure 1:**
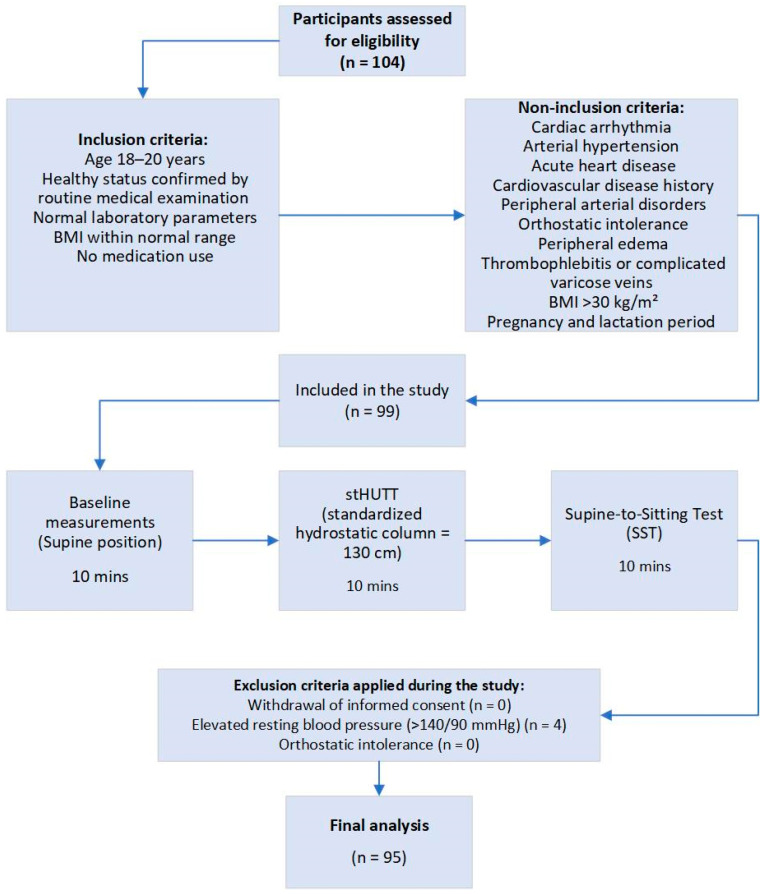
Flowchart of the study design and patient selection.

The research was conducted at Smolensk State Medical University and at Federal Research and Clinical Center of Intensive Care Medicine and Rehabilitology in 2024–2025 (Table 1, Figure 1).

Inclusion criteria: healthy participants aged between 18 and 20; who had undergone a routine medical examination with objective laboratory parameters within normal ranges, body mass index 21.3 [19.2; 24] kg/m^2^; blood pressure 100–130/75–85 mm Hg; no history of taking any medication. Participants were advised to refrain from smoking and alcohol consumption for two days prior to the study, as well as to avoid coffee and strenuous physical activity 24 h before the measurements.

Non-inclusion criteria: cardiac arrhythmia, arterial hypertension, acute heart disease, history of cardiovascular disease, peripheral arterial blood flow disorders, history of orthostatic intolerance, peripheral edema, pregnancy or lactation, signs of thrombophlebitis or complicated varicose veins, body mass index > 30 kg/m^2^.

Data from four subjects were excluded from the analysis, as their resting blood pressure in the supine position exceeded 140/90 mmHg.

### 2.2. Orthostatic Tests Procedure

The study was divided into three stages: rest in a supine position (baseline), the stHUTT at an individual tilt angle ensuring a standard hydrostatic column height of 130 cm—the Luanda protocol—and the subjects’ active transition to a sitting position. Each stage lasted 10 min. We did not apply the extended study protocol, which included a return to the baseline position after the stHUTT and after the sitting position, as in our previous studies on the same cohort using the stHUTT, all parameters returned to baseline values upon returning to the baseline position [35]. After the stHUTT, the subject was seated on a standard chair with a seat height of 46 cm and a straight backrest. Sitting and standing height were measured. Depending on their height, the subjects exhibited a knee flexion angle of 110° or more.

### 2.3. Haemodynamic Measurements

All measurements of systolic blood pressure (SBP), diastolic blood pressure (DBP) and brachial–ankle pulse wave velocity (baPWV) were performed using a multichannel sphygmomanometer (ABI-System 100 PWV, BOSO, Berlin, Germany) after 10 min of rest in each of the three positions: the baseline position, the stHUTT and the sitting position. The measurements were taken three times, the first measurement was excluded, and the second and third measurements were averaged. The reference side was defined as the arm with the higher systolic blood pressure (SBP), and all subsequent measurements were recorded on that side. In addition, calculations for FBM-VS: the functional reserve of orthostatic blood circulation regulation (FR) during stHUTT (FR_st_) and during the supine-to-sitting test (FR_sit_), and the FR coefficients in the same tests (FRC_st_ and FRC_sit_) were performed.

FR_st_ = (baPWV_st_ − baPWV_b_)

FR_sit_ = (baPWV_sit_ − baPWV_b_)

FRC_st_ = (baPWV_st_ − baPWV_b_)/baPWV_b_ = FR_st_/baPWV_b_

FRC_sit_ = (baPWV_sit_ − baPWV_b_)/baPWV_b_ = FR_sit_/baPWV_b_

where baPWV_b_ is baPWV at baseline (supine position), baPWV_st_ is baPWV in the stHUTT position, and baPWV_sit_ is baPWV in the sitting position [31].

### 2.4. Statistical Analysis

For data collection, adjustment, and systematization, as well as for visualization of the results, Microsoft Excel 2021 and STATISTICA 10 (TIBCO Software, Palo Alto, CA, USA) were used. Nominal data were presented as absolute values. Quantitative variables without a normal distribution were described using the median and quartiles (25th–75th percentiles, Q1–Q3). The Kolmogorov–Smirnov test was applied to assess the distribution pattern. To describe the variability of functional biomarkers obtained during the stHUTT and in supine-to-sitting test, variance (σ^2^) was additionally calculated. Comparisons of dependent samples (paired observations) were performed using the Wilcoxon signed-rank test. The relationships between the indices obtained during both tests were assessed using Spearman rank correlation analysis. Agreement and systematic bias between SST and stHUTT-derived biomarkers were additionally assessed using Bland–Altman analysis. Mean bias and 95% limits of agreement were calculated. A post hoc power analysis was performed using G*Power 3.1 software. Based on rs = 0.677, n = 95 and α = 0.05, the achieved statistical power was 0.999. A *p*-value < 0.05 was considered statistically significant.

## 3. Results

In accordance with the primary objective of this study, we compared brachial blood pressure (BP) and functional biomarkers of vascular stiffness (FBM-VS) across body positions: stHUTT and SST (Table 2).

Among the study participants, baseline SBP and DBP values, along with baPWV measurements, were within age-appropriate normal ranges [36]. During the stHUTT and SST, SBP decreased while remaining within the normal range; the decrease was less pronounced in the sitting position (*p* < 0.001). DBP increased significantly in both tests compared with baseline, with the increase being more pronounced in the sitting position compared with stHUTT (*p* < 0.001), whereas HR increased in both tests, with a more pronounced increase during stHUTT compared with SST (*p* < 0.001) (Table 2). During both functional tests baPWV increased significantly compared with baseline values, from 8.6 [8.1; 9.1] m/s to 15.2 [13.4; 16.1] m/s during SST (*p*_1-3_ < 0.001), compared with 13.4 [12.1; 14.4] m/s during stHUTT (*p*_2-3_ < 0.001). Functional biomarkers were also higher during SST than during stHUTT (FRsit = 6.4 [5.25; 7.75] m/s vs. FRst = 4.85 [3.7; 5.75] m/s (*p*_2-3_ < 0.001); FRCsit = 0.74 [0.59; 0.9] vs. FRCst = 0.55 [0.45; 0.68], (*p*_2-3_ < 0.001).

**Figure 2:**
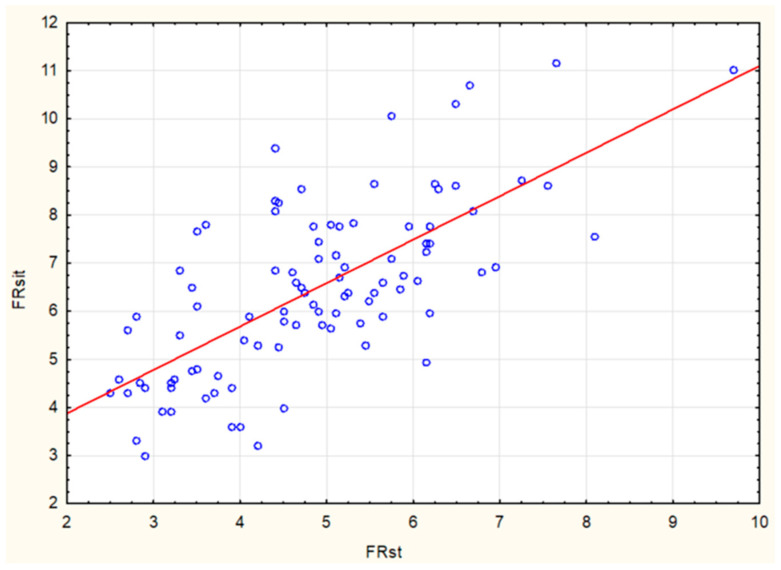
Spearman rank correlation analysis of paired measurements of functional reserve (FR) obtained during the stHUTT and the simplified SST. Blue circles denote individual observations; the red line represents the linear regression trend.

**Figure 3:**
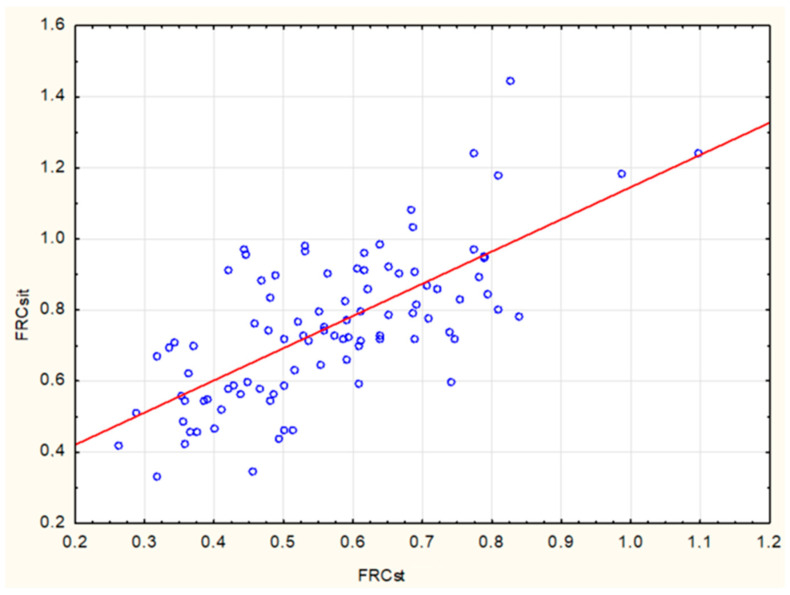
Spearman rank correlation analysis graph between paired measurements of functional reserve coefficient (FRC) obtained during the stHUTT and the simplified SST. Blue circles denote individual observations; the red line represents the linear regression trend.

In addition, we performed a correlation analysis of biomarkers measured using both tests in paired samples (Figure 2 and Figure 3).

The graph demonstrates the Spearman rank correlation analysis of FR values in both tests. A strong positive association was observed between the values obtained in the simplified SST and in the stHUTT, with a correlation coefficient of r = 0.676 (*p* < 0.0001).

A comparison of FRC values calculated during the stHUTT test and the simplified SST also revealed a strong correlation (r = 0.673, *p* < 0.0001).

The stHUTT protocol was developed to minimize the influence of height variability on the outcomes of these measurements. In our study, to evaluate the effectiveness of mitigating this factor, we conducted a quantitative assessment of the variance of FBM-VS measured during stHUTT and the simplified SST (Table 3).

A comparative analysis of FBM-VS variability revealed a significant increase in biomarker variability during the simplified SST: baPWV variability increased from 2.86 during the stHUTT to 4.28 during simplified SST, FR from 1.92 to 3.19, and FRC from 0.02 to 0.04. We attribute the increased variability in functional biomarker values during simplified SST to the greater influence of differences in hydrostatic column heights—which depend on the subjects’ height—as opposed to the standard hydrostatic load used in stHUTT. This is of great importance for selecting an appropriate research protocol when testing related and unrelated samples.

Bland–Altman analysis demonstrated a mean bias of 1.60 m/s for FR, with 95% limits of agreement ranging from −0.91 to 4.11 m/s. For FRC, the mean bias was 0.187, with 95% limits of agreement ranging from −0.106 to 0.479.

## 4. Discussion

Prediction plays a pivotal role in the prevention of chronic diseases. Currently, the prediction of arterial hypertension and other age-related vascular diseases is primarily based on monitoring population-level predictors and risk factors. While acknowledging the undeniable value of this approach, it is equally important to implement individualized monitoring of risk markers that may be pathogenetically linked to the development of these conditions and capable of increasing all-cause mortality, thereby serving as key indicators of biological age [21,37,38,39]. Monitoring such markers at the preclinical stage of chronic disease development in apparently healthy population groups may have the greatest clinical value. In our earlier pilot study investigating age-related changes in FBM-VS and parameters of vascular stiffness measured at rest in the supine position, we identified divergent trends: an age-related increase in vascular stiffness accompanied by a progressive age-related decline in FBM-VS, with peak values observed in young, healthy subjects [31,32]. It is well known that in the development of chronic diseases, functional deviations precede clinical manifestations. Accordingly, we hypothesized that an accelerated decline in FBM-VS precedes the structural remodeling of the vascular wall, leading to an increase in traditionally measured vascular stiffness. Age-related increases in arterial stiffness are a widely recognized indicator of vascular aging, which can progress according to three different scenarios: normal, accelerated, and decelerated [40,41,42]. Accelerated vascular aging significantly increases the risk of developing age-related vascular diseases and accelerates target organ damage. Therefore, it is important to detect this phenotype of vascular aging at an early stage, even in a healthy population, which should help improve the effectiveness of primary prevention of ARVD. Regular, population-wide monitoring of FBM-VS, starting from a young age, could become one of the possible diagnostic strategies for the early detection of an unfavorable phenotype of vascular aging.

For the potential population-wide personalized screening of the risk of accelerated arterial stiffness increase in healthy populations to effectively prevent cardiovascular diseases, we developed a simplified SST protocol for the quantitative assessment of FBM-VS, applicable in both clinical and home settings. Previously, using stHUTT, we demonstrated the feasibility of quantitatively measuring FBM-VS and detecting preclinical functional deviations in healthy subjects [32]. This method has proven effective in comparative studies of independent samples for assessing racial, sex, and age differences. In the present study, by comparing hemodynamic parameters in the same subjects during stHUTT and SST, we aimed to develop a screening tool for assessing orthostatic blood flow regulation, allowing for personalized evaluation of its changes and dynamics long before the onset of structural vascular changes and clinical manifestations of ARVD. The application of SST allowed us to simultaneously address two objectives: personalizing the hydrostatic load proportional to the subjects’ height, and significantly reducing the cost of the test by using only an examination couch and a standard chair. For repeated longitudinal measurements, it is sufficient to reproduce the same hydrostatic column height, i.e., the vertical distance from the foot support plane to the chair seat surface. Thus, the need for a bulky tilt table was eliminated.

To assess the applicability of the simplified SST, the need for which was justified above, we compared the measurement results obtained during stHUTT and SST. In the sitting position, SBP and DBP values were significantly higher than during stHUTT, which is consistent with the results of a study using a comparable protocol in a similar cohort of young individuals [43]. The functional biomarkers FR and FRC were statistically significantly higher during SST compared to stHUTT. Thus, we observe that SST in our study elicits a more pronounced hemodynamic response compared to stHUTT. Presumably, the magnitude of the response to the orthostatic test is influenced by the height of the hydrostatic column, which is 130 cm for stHUTT, whereas during SST it depends on the subject’s height measured in the sitting position (134 [131; 138] cm). In addition, the sitting leg position likely has an effect. Therefore, their optimal position during SST was defined (‘the subjects exhibited a knee flexion angle of 110° or more’), which should also be maintained during subsequent longitudinal studies.

Literature data regarding the comparison of vascular stiffness in the supine and sitting positions are contradictory. Some authors report an increase in arterial stiffness, measured by pulse wave velocity (PWV), in the sitting position [44]. Others find no statistically significant differences in PWV values between the supine and sitting positions [45]. It should be noted that in both cases, the measurement involved carotid-femoral pulse wave velocity (cfPWV), which predominantly assesses the stiffness of the aorta and large elastic-type arteries, where the content of smooth muscle cells mediating responses to neurohormonal changes is minimal. In contrast, our study measured brachial–ankle pulse wave velocity (baPWV), which also encompasses muscular-type arteries. In such vessels of the legs, hydrostatic pressure increases substantially during orthostatic challenges, thereby eliciting a more pronounced physiological response.

We observed unidirectional changes in FR and FRC in both orthostatic tests, with a strong Spearman rank correlation between the paired samples. At the same time, group-level analysis revealed significantly greater variability in FR and FRC during the simplified SST compared to stHUTT. This is attributable to the differences in hydrostatic column heights among subjects during SST, related to individual differences in height, and the absence of such differences during stHUTT, given that the hydrostatic column height modulates the magnitude of the adaptive neurohormonal shift during the orthostatic test.

The stHUTT protocol proves to be more effective for both cross-sectional and longitudinal population-based FBM-VS studies in independent cohorts. Standardized hydrostatic loading enables more precise analysis of intergroup differences in response to an identical physiological stimulus. Conversely, SST is inherently more personalized, as it is directly dependent on individual anthropometric data. The advantages of SST include operational simplicity, allowing its application in both clinical and home settings provided appropriate equipment is available, as well as the personalization of the hydrostatic load based on human height, linking it to the hydrostatic load experienced in daily life.

The application of SST for the dynamic assessment of FBM-VS during repeated measurements requires strict reproduction of test conditions, which can be achieved by reproducing the required vertical height between the foot support plane and the chair seat, as well as the required leg position. For repeated FBM-VS measurements during longitudinal follow-up, the required height can be reproduced either by adjusting the chair seat height or, more conveniently, by modifying the height of the footrest, thereby ensuring a constant vertical distance from the support surface to the vertex of the head across all sessions. Assessments are preferably conducted in the morning, 1–2 h after a light breakfast, to minimize the potential effects of circadian rhythms and psychoemotional status on the neurohormonal background.

The simplified FBM-VS assessment protocol was developed for the personalized quantitative analysis of age-related dynamics during longitudinal monitoring of functional changes, as well as for evaluating the effectiveness of interventions aimed at slowing down vascular aging.

### 4.1. Study Limitations

One of the limitations of this study is the narrow age range of the sample, which consisted of young healthy subjects aged 18–20 years, limiting the generalizability of the results to other age categories and complicating the assessment of the prognostic value of dynamic FBM-VS analysis. This choice was driven by the aim of our study—a comparative analysis of the application of stHUTT and a simplified SST for measuring FBM-VS in a paired sample of healthy young subjects—and the need to develop a screening tool for assessing orthostatic blood flow regulation, allowing for personalized evaluation of its changes and dynamics long before the onset of structural vascular changes and clinical manifestations of ARVD.

Currently, there are no age-specific reference values for FBM-VS during SST derived from large samples. However, we have previously shown that functional biomarkers of vascular stiffness decrease significantly with age. From a clinical perspective, when analyzing biomarkers of vascular stiffness, the focus should not be on single measurements but on the dynamics of their values in the same individual, measured under identical test conditions.

The aforementioned limitations will be addressed in upcoming prospective cohort studies of age-related FBM-VS dynamics in healthy populations.

Furthermore, we believe that for population-based studies of independent samples differing in race, sex, geography, etc., the use of stHUTT rather than SST is more appropriate, as it provides a standard hydrostatic column height.

### 4.2. Perspectives

We hypothesise that, within the framework of P4 medicine, periodic assessment of FBM-VS could become a sensitive tool for analyzing the individual dynamics of vascular aging and the state of the cardiovascular system. Therefore, in the future, such a tool could be routinely used during regular preventive health examinations of healthy individuals for the timely evaluation of the effectiveness of selected individual prevention strategies. In patients with ARVD, through the objective assessment of the impact of interventions on FBM-VS dynamics, this tool could potentially become one of the criteria for optimizing treatment, taking into account the need to increase FBM-VS or, at the very least, prevent their rapid decline.

## 5. Conclusions

The supine-to-sitting test may be used for the personalized diagnostic assessment of functional biomarkers of vascular stiffness in healthy populations. To assess their prognostic value and to provide personalized long-term monitoring to control the effectiveness of preventive measures against vascular ageing in healthy individuals, a prospective cohort study is required.

## Figures and Tables

**Table 1:** The characteristics of young adults.

Participants	*N* = 95
Sex (Male/Female)	41/54
Age (years)	18 [18; 20]
Height (cm)	175 [168.5; 181]
Sitting Height (cm)	134 [131; 138]
Body mass index (kg/m^2^)	21.3 [19.2; 24]

Note: The data are presented as Me [range]—median—for parameters that were not normally distributed, quartiles—[25%; 75%]—margins of the interquartile range.

**Table 2:** Hemodynamic parameters and vascular stiffness markers in young healthy subjects according to body position.

Parameters	Baseline	stHUTT (st)	SST (sit)	*p*
	1	2	3	
SBP (mmHg)	118 [114; 122]	111 [103; 118]	117 [112; 122]	*p*_1-2_ < 0.001 *p*_2-3_ < 0.001 *p*_1-3_ = 0.79
DBP (mmHg)	69.5 [66; 73.5]	72 [66; 77]	77 [73.5; 80.5]	*p*_1-2_ = 0.003 *p*_2-3_ < 0.001 *p*_1-3_ < 0.001
HR (b/min)	66 [62; 73]	78 [71; 84]	73 [66; 79.5]	*p*_1-2_ < 0.001 *p*_2-3_ < 0.001 *p*_1-3_ < 0.001
baPWV (m/s)	8.6 [8.1; 9.1]	13.4 [12.1; 14.4]	15.2 [13.4; 16.1]	*p*_1-2_ < 0.001 *p*_2-3_ < 0.001 *p*_1-3_ < 0.001
FR (m/s)	-	4.85 [3.7; 5.75]	6.4 [5.25; 7.75]	*p*_2-3_ < 0.001
FRC		0.55 [0.45; 0.68]	0.74 [0.59; 0.9]	*p*_2-3_ < 0.001

Note: SBP, systolic blood pressure brachial; DBP, diastolic blood pressure brachial; HR, heart rate; baPWV, brachial–ankle pulse wave velocity; FR, functional reserve; FRC, functional reserve coefficient. Values are presented as median [interquartile range]. Subscripts denote measurement condition: st, stHUTT; sit, SST. Intergroup differences were considered significant at *p* < 0.05. The *p*-value was calculated using the Friedman test for dependent samples. SBP: χ^2^(2) = 50.8; *p* < 0.001; W = 0.27. DBP: χ^2^(2) = 67.5; *p* < 0.001; W = 0.35. HR: χ^2^(2) = 99.7; *p* < 0.001; W = 0.52. baPWVb: χ^2^(2) = 173.3; *p* < 0.001; W = 0.91.

**Table 3:** Quantitative assessment of the variance (σ^2^) of functional biomarkers during the stHUTT and during the SST.

Parameters	stHUTT (st)	SST (sit)
σ^2^(baPWV) m^2^/s^2^	2.86	4.28
σ^2^(FR) m^2^/s^2^	1.92	3.19
σ^2^(FRC)	0.02	0.04

Note: σ^2^, variance; baPWV, brachial–ankle pulse wave velocity; FR, functional reserve; FRC, functional reserve coefficient. Subscripts denote measurement condition: st, stHUTT; sit, SST.

## Data Availability

The data that support the findings of this study are available from the corresponding author, Victor N. Dorogovtsev, upon reasonable request.

## Abbreviations

The following abbreviations are used in this manuscript:

AH	Arterial Hypertension
ARVDs	Age-Related Vascular Diseases
baPWV	brachial–ankle Pulse Wave Velocity
BP	Blood Pressure
cfPWV	Carotid–Femoral Pulse Wave Velocity
DBP	Diastolic Blood Pressure
FBM-VS	Functional Biomarkers of Vascular Stiffness
FR	Functional Reserve
FRC	Functional Reserve Coefficient
P4 Medicine	Predictive, Preventive, Personalised and Participatory Medicine
SBP	Systolic Blood Pressure
sit	Sitting position
st	Standardized head-up tilt position
SST	Supine-to-Sitting Test
stHUTT	Standardized to hydrostatic column height Head-Up Tilt Test
